# Techniques for Non-Invasive Monitoring of Arterial Blood Pressure

**DOI:** 10.3389/fmed.2017.00231

**Published:** 2018-01-08

**Authors:** Agnes S. Meidert, Bernd Saugel

**Affiliations:** ^1^Department of Anaesthesiology, University Hospital, Ludwig-Maximilians-Universität München, Munich, Germany; ^2^Department of Anesthesiology, Center of Anesthesiology and Intensive Care Medicine, University Medical Center Hamburg-Eppendorf, Hamburg, Germany

**Keywords:** blood pressure monitoring, perioperative monitoring, non-invasive blood pressure, arterial pressure, oscillometry, applanation tonometry, vascular unloading technique

## Abstract

Since both, hypotension and hypertension, can potentially impair the function of vital organs such as heart, brain, or kidneys, monitoring of arterial blood pressure (BP) is a mainstay of hemodynamic monitoring in acutely or critically ill patients. Arterial BP can either be obtained invasively via an arterial catheter or non-invasively. Non-invasive BP measurement provides either intermittent or continuous readings. Most commonly, an occluding upper arm cuff is used for intermittent non-invasive monitoring. BP values are then obtained either manually (by auscultation of Korotkoff sounds or palpation) or automatically (e.g., by oscillometry). For continuous non-invasive BP monitoring, the volume clamp method or arterial applanation tonometry can be used. Both techniques enable the arterial waveform and BP values to be obtained continuously. This article describes the different techniques for non-invasive BP measurement, their advantages and limitations, and their clinical applicability.

## Background

Monitoring of arterial blood pressure (BP) is a mainstay of hemodynamic monitoring in acutely or critically ill patients. Close monitoring of BP is of great importance to detect and treat hypotension and hypertension early. Both, hypotension and hypertension can impair the function of vital organs, such as the brain (1), the heart (2), and the kidneys (3).

The direct measurement of BP via arterial cannulation is regarded as the clinical reference method (criterion standard). In clinical routine, it is commonly performed during high-risk surgery and in intensive care medicine. The cannulation of an artery, however, can be time-consuming, needs to be done by a trained operator, and is associated—although very rarely (4)—with potential major complications such as embolism, lesion of nerves or vessels, or ischemia. For these reasons, BP is very commonly measured non-invasively.

**Figure 1:**
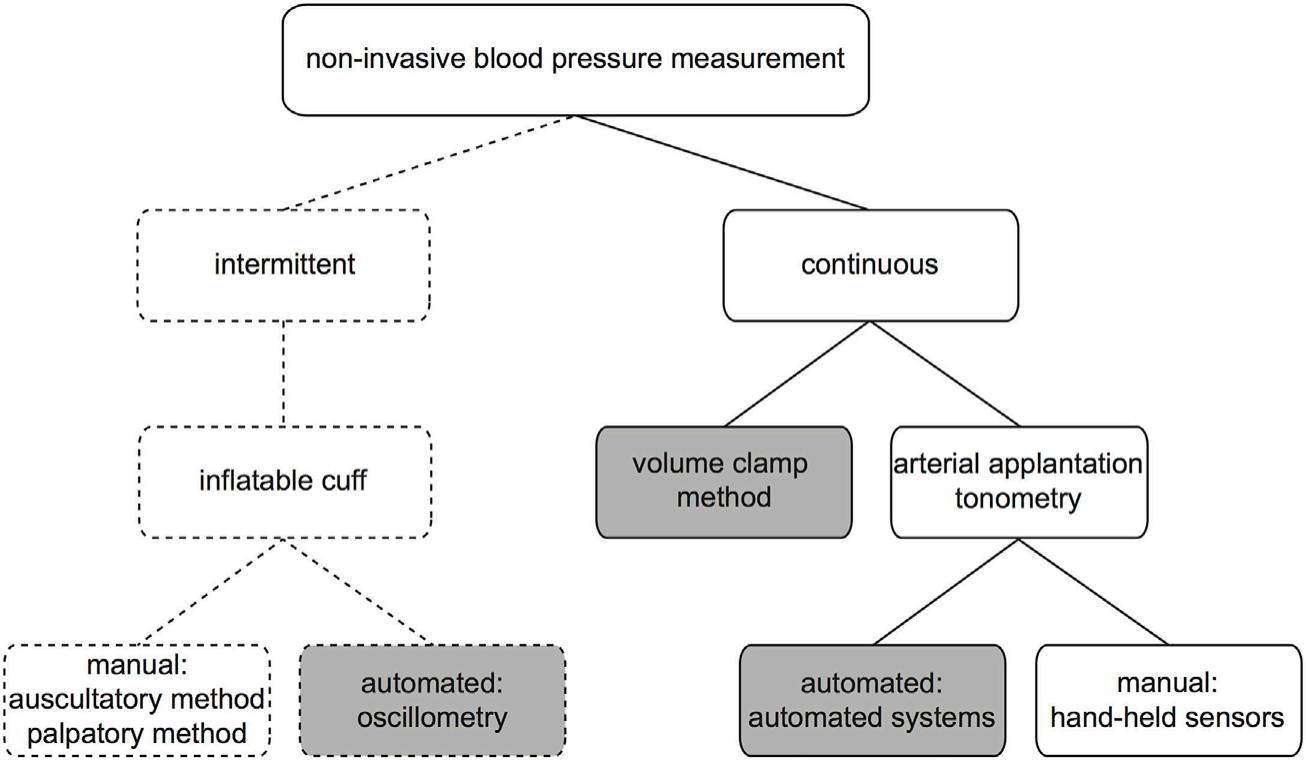
Non-invasive blood pressure monitoring techniques.

There are several ways to non-invasively measure BP. Monitoring techniques can be classified according to their ability to measure BP intermittently or continuously (Figure 1). In this article, we describe techniques for non-invasive monitoring of arterial BP and discuss their advantages, limitations, and clinical applicability.

## Non-Invasive Intermittent Techniques

For intermittent BP measurement, an air-filled occluding cuff can be used that enables BP to be measured either manually or automatically. For all occluding cuffs, the right size is critical for valid measurement (5). Manual measurement of BP by an occluding cuff can be done either by palpation or auscultation (6).

With the palpatory method, an inflatable cuff is wrapped around the upper arm of a patient. The manometer connected to the cuff by a tube shows the pressure applied. The physician feels the radial pulse, inflates the cuff until the brachial artery collapses, and there is no blood flow any more. The pressure at which a pulse can be detected again while deflating the cuff corresponds to the systolic arterial pressure of the patient. This method does not need a stethoscope or any other specific skills or equipment and can also be performed in a noisy environment. However, it only provides the systolic arterial pressure. The auscultatory method is performed in a similar way; after inflation of the cuff to a pressure above the systolic pressure (verified by the vanished radial pulse), the typical Korotkoff sounds can be detected by a stethoscope applied distal of the upper arm cuff during slow deflation. The onset of the sounds corresponds to the patients’ systolic arterial pressure, the last sound at decreasing cuff pressure equals the patients’ diastolic arterial pressure. The advantage of this technique is that it provides the diastolic arterial pressure value, disadvantages include the need for training how to correctly apply this technique and the need of a stethoscope and a quiet environment.

An automated method to measure BP with the help of an occluding cuff employs the oscillometric technique. The cuff is inflated to a preset value automatically. Then, the pressure is gradually being reduced. The pressure wave causes oscillations in the vessel, which can be detected by the cuff. Mean arterial pressure corresponds to the maximum of oscillations (7); an algorithm applied to the change of oscillations sets systolic and diastolic arterial pressure values. These proprietary algorithms differ between manufacturers and are often not publicly available (8). The advantages of oscillometry are mainly the presence of reasonably accurate mean arterial pressure (in normal BP ranges) and the possibility of having an automated tool to determine a patient’s BP at a preset interval. The disadvantages are the overestimation of low and underestimation of high values (9, 10) and the possibility to falsify measurements [e.g., by movement (detected as oscillations) or the patient’s arm resting on the bed] (11).

The intermittent nature of BP measurements provided by all the techniques described earlier is a disadvantage they all have in common.

## Non-Invasive Continuous Techniques

During the recent years, continuous non-invasive BP monitoring techniques became available that enable a real-time BP curve and numerical BP values to be assessed (just with direct BP measurement).

The continuous non-invasive measurement principles are based on either one of two different techniques, namely arterial applanation tonometry or the volume clamp method. Arterial applanation tonometry is based on the work of Pressman and Newgard (12), who found that a transducer strapped to an artery with a bone underneath, can obtain the arterial pulse wave. The technique has been refined and now is able to assess mean arterial pressure in the radial artery and allows the calculation of diastolic and systolic arterial pressure (e.g., using population-based algorithms) (13). The technique is used in cardiology to assess central vascular pressures (14). The pulse wave obtained by applanation tonometry can be analyzed and bears more information than systolic and diastolic pressure alone. However, these devices are not made for continuous patient monitoring as they have to be hand held by the examiner. A device allowing automated radial artery applanation tonometry is the T-Line system (Tensys Medical, San Diego, CA, USA) (15, 16). The system has been evaluated in various clinical settings (13, 16–20).

The second technique for non-invasive continuous BP measurement is called volume clamp method (or vascular unloading technology) based on the work by Penaz et al. (21). The BP is measured at the finger with an inflatable cuff combined with a photodiode. The diameter of the artery in the finger is measured by the photodiode; the pressure in the cuff is adjusted to keep the diameter of the artery constant. From the pressure changes in the cuff, a BP curve can be calculated and transferred to correspond to brachial artery BP. Devices based on this technique are ClearSight (Edwards, Irvine, CA, USA) and CNAP (CNSystems Medizintechnik AG, Graz, Austria).

The continuous non-invasive devices are all sensitive to patient movement; therefore, monitoring of the conscious patient is possible but measurement results need to be checked for plausibility. In case of severe vasoconstriction, peripheral vascular disease, or distorted fingers due to arthritis, clinical experience has shown that it may be difficult to obtain a valid waveform using finger cuffs. Some patients report discomfort from the congestion in venous return from the fingertip where the cuff is place. For this reason, manufacturers recommend to change the cuff to another finger after a certain period of monitoring. In addition, compared to conventional intermittent devices for BP measurement, continuous BP monitoring is relatively expensive.

## Are the Techniques Reliable?

Most clinicians ask themselves, whether the non-invasively obtained BP curve shows the “real” BP. Therefore, it is inevitable to discuss the measurement performance in terms of accuracy and precision of the various non-invasive devices. The pressure measured within an artery by means of arterial cannulation is regarded as the reference method of BP measurement. In the absence of direct BP measurement, the auscultatory method with a mercury column is regarded as the “gold standard.” However, as Alpert and colleagues (11) point out, the cuff/stethoscope method itself sometimes differs considerably from intra-arterial pressure. Nonetheless, when evaluating a new non-invasive device using an occluding cuff, reference measurements are performed by the auscultatory method (22). This has led to the common belief that the upper arm cuff measurements represent the “real” BP of a patient. Since the auscultatory method is now widely replaced by devices that engage an oscillometric technology clinicians trust the values produced by the device with the upper arm cuff (23). A survey by Chatterjee and colleagues (23) showed that even in critically ill patients on vasopressors the non-invasive upper arm BP measurement was used to guide therapy by 47% of respondents, although intensivists would be expected to know about the limitations of oscillometric measurement in unstable patients. However, big data base analyses of simultaneous measurements on ICU an OR have demonstrated that the devices using an oscillometric method tend to overestimate hypotensive BP values and to underestimate hypertensive BP values (9, 10). Within the normal BP range, the measurement of mean arterial pressure seems to be sufficiently accurate (9, 10, 24). Studies on the accuracy of oscillometric mean arterial BP in critically ill patients demonstrated that a possible source for inaccuracy lies within the choice of the wrong cuff size (25, 26). However, even when the correctly sized cuff was used, the results still showed clinically unacceptable discrepancy between invasive and non-invasive values (25, 26). Focusing on a possible relationship between obesity and inaccuracy of non-invasive BP measurement, Araghi et al. (27) studied overweight patients in the ICU. The analysis revealed clinically relevant inaccuracy of both, auscultatory and oscillometric, techniques (27), which therefore should not be used to guide therapy in critically ill patients. A similar study compared invasive and oscillometric BP measurement in obese patients undergoing non-cardiac surgery (28). In addition, the same group also examined the cuff position at the forearm of patients. However, oscillometric measurement in these patients in both locations did not allow sufficiently accurate monitoring of BP (28). This leads to the question, whether BP values resulting from oscillometric measurement with other locations for the cuff than the upper arm can be used for guidance of therapy. A single-center study in ICU patients showed acceptable agreement for oscillometric mean arterial BP compared to intra-arterial BP when the cuff was placed at the upper arm, whereas the thigh and ankle location revealed inaccurate values (29). In accordance to these findings, Drake and Hill (30) performed upper arm and ankle measurements during elective cesarean section. The values from the different sites varied considerably; therefore, the oscillometric measurement at the ankle cannot be seen as an alternative to the upper arm (30).

The reliability of non-invasive intermittent BP measurement in patients with arrhythmia has been questioned (31). Two studies have shown recently that there is no relevant difference between oscillometric measurement in patients with or without arrhythmia (32, 33).

For all non-invasive devices that measure BP continuously, numerous validation studies exist (34–36). Kim et al. (35) pooled data from various studies comparing non-invasive continuous devices with direct BP, which have been published until 2013 and reported the mean of the differences with its SD. By this approach, they found an overall random-effect pooled bias for mean BP of 3.2 ± 8.4 mmHg. When stratifying the results according to the different measurement technologies described earlier, the analysis for mean BP yielded a bias and SD of 1.3 ± 5.7, 5.5 ± 9.3, and 3.5 ± 6.8 mmHg for the T-Line system, CNAP, and ClearSight, respectively (35). This analysis demonstrated that accuracy and precision of continuous non-invasive devices are not interchangeable with invasive BP measurement. Besides, the group criticized the lack of a recognized standard to define clinical acceptability (35). Vos et al. (37) concluded recently that non-invasive continuous monitoring with ClearSight was interchangeable with monitoring by an oscillometric technique. In their review from 2016, Bartels and colleagues (34) relate these findings to the well-known inaccuracy of oscillometry. The question is whether continuous non-invasive devices need to replace the direct measurement or rather fill the monitoring gap for patients who are insufficiently monitored by intermittent measurements only. Some clinicians find the ability to track changes in BP of the continuous devices particularly helpful in managing patient care.

In the end, the operator has to know about the limitations and pitfalls of any BP measuring technique, both non-invasive and invasive, to select the optimal technology for BP monitoring for the individual patient.

## How Should We Measure BP?

The BP monitoring that we use for the individual patient needs to be tailored to the needs of the patients and the clinical setting.

For critically ill patients in the ICU, non-invasive BP monitoring is unlikely to play a big role in the foreseeable future. Although some researchers see the age of total non-invasive BP monitoring dawning (38), in our point of view critically ill patients need frequent arterial blood gas analysis as well as continuous and reliable measurement of absolute BP values.

In the emergency department, it is crucial to identify hemodynamic instability early. Intermittent BP monitoring, however, often is set at quite long intervals (e.g., 15 or 30 min) resulting in missing or only delayed detection of hypotension. There are studies that point out the advantage of continuous monitoring in terms early recognition of deterioration of the patient’s hemodynamic status (39, 40).

For patients undergoing surgical procedures, the appropriate method of BP monitoring needs to be identified considering perioperative cardiovascular risk stratification. There are different types of hypotension during general anesthesia and surgery, e.g., post-induction hypotension, early intraoperative hypotension, and late intraoperative hypotension with different risk factors (41). There is a growing body of evidence that continuous monitoring can be beneficial in terms of BP stability. As Walsh et al. (3) showed even periods of hypotension as short as a few minutes can adversely affect organ function. Therefore, BP measurement should first of all enable the physician to maintain BP stability in the patient. Benes and colleagues (42) have demonstrated that continuous BP measurement helps to keep the BP stable during surgery in beach chair positioning compared to intermittent measurements taken every 5 min. Recently, it has been shown in 160 patients with a history of hypertension that there are significantly less hypotensive episodes during induction of general anesthesia when a continuous method is used instead of intermittent oscillometric measurements every 3 min (43). For patients undergoing planned cesarean section, continuous monitoring helped to detect hypotensive episodes earlier and more often (44).

**Figure 2:**
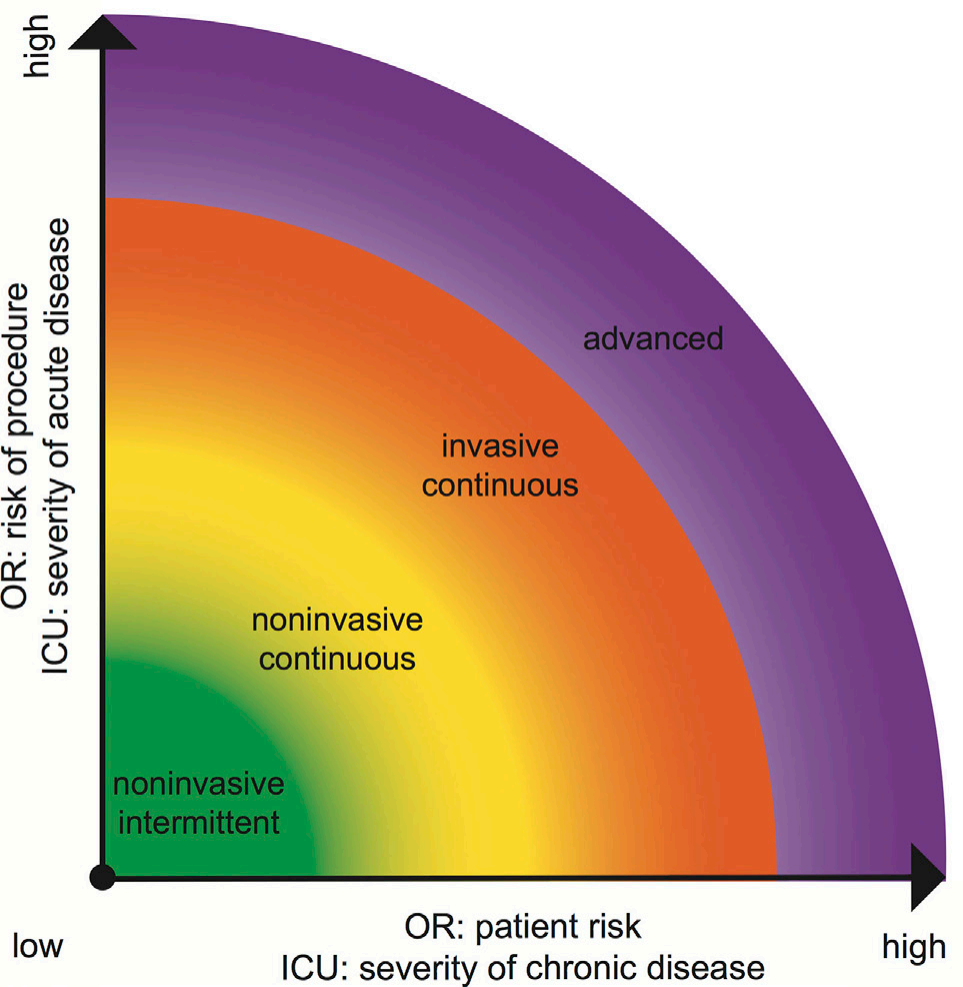
Choice of adequate blood pressure monitoring equipment in ICU and OR according to patient and procedural risk (OR) (45) or chronic and acute disease (ICU).

In the perioperative setting, the likelihood for intraoperative hypotension and the patient’s risk to develop hypoperfusion-induced organ failure should lead to the choice of which BP monitoring to use (Figure 2). We recommend advanced hemodynamic monitoring that allows monitoring of blood flow and fluid responsiveness parameters in the OR for high risk patients undergoing high-risk procedures (45) and in the ICU for patients with severe chronic and acute disease (Figure 2).

## Conclusion

Blood pressure monitoring needs to be tailored to the individual patient. In stable, low-risk patients, intermittent oscillometric BP measurements are usually sufficient. Patients who are at risk for hemodynamic instability should be monitored by continuous BP measurement. Whether continuous non-invasive BP monitoring can improve patient outcome in certain patient collectives or clinical settings (perioperative medicine, emergency medicine) is the subject of the current clinical research. In critically ill patients, we still recommend the continuous invasive BP monitoring with an arterial catheter.

## Author Contributions

All authors listed, have made a substantial, direct and intellectual contribution to the work, and approved it for publication.

## Conflict of Interest Statement

AM has no conflict of interest. BS collaborates with Pulsion Medical Systems SE (Feldkirchen, Germany) as a member of the medical advisory board and received honoraria for giving lectures and refunds of travel expenses from Pulsion Medical Systems SE. BS received research support from Edwards Lifesciences (Irvine, CA, USA). BS received institutional research grants, unrestricted research grants, and refunds of travel expenses from Tensys Medical Inc. (San Diego, CA, USA). BS received honoraria for giving lectures and refunds of travel expenses from CNSystems Medizintechnik AG (Graz, Austria).
